# Is Embodied Cognition Bilingual? Current Evidence and Perspectives of the Embodied Cognition Approach to Bilingual Language Processing

**DOI:** 10.3389/fpsyg.2019.00108

**Published:** 2019-02-06

**Authors:** Katharina Kühne, Claudia Gianelli

**Affiliations:** Division of Cognitive Sciences, University of Potsdam, Potsdam, Germany

**Keywords:** embodied language, grounding, sensorimotor, native language, second language

## Abstract

Accumulating behavioral and neurophysiological evidence supports the idea of language being grounded in sensorimotor processes, with indications of a functional role of motor, sensory and emotional systems in processing both concrete and abstract linguistic concepts. However, most of the available studies focused on native language speakers (L1), with only a limited number of investigations testing embodied language processing in the case of a second language (L2). In this paper we review the available evidence on embodied effects in L2 and discuss their possible integration into existing models of linguistic processing in L1 and L2. Finally, we discuss possible avenues for future research towards an integrated model of L1 and L2 sensorimotor and emotional grounding.

## Introduction

Amodal system theories of language suggest that cognition is built of abstract amodal representations via formal rules, related to their referents (Newell and Simon, 1972; Collins and Loftus, 1975). These theories presuppose that lower-level processes of perception and action play no role in forming cognition (Ojemann, 1991). This assumption has recently been challenged by studies demonstrating that neural sensorimotor systems are also active during language processing (Buccino et al., 2001; Hauk et al., 2004; Hauk and Pulvermüller, 2004; Shtyrov et al., 2004; Pulvermüller et al., 2005; Tettamanti et al., 2005; Sakreida et al., 2013), suggesting that language is grounded in bodily action and perception, or ‘embodied’. Numerous studies have demonstrated how processing linguistic items with sensory, motor and emotional content re-activate the same neural structures as the experience of that content (e.g., executing a certain action, or feeling a certain emotion). Noteworthy, while most research has so far focused on how concrete concepts are grounded in sensorimotor processes, there is evidence suggesting that also more abstract concepts might be similarly grounded (for review see Fischer and Zwaan, 2008; Pulvermüller and Fadiga, 2010; Buccino et al., 2016).

Despite the accumulating evidence, however, the exact functional role of these activations remains debated. One possibility is that embodied mechanisms are indeed an inseparable and functionally crucial part of language processing (Vukovic et al., 2017). Oppositely, they might represent just a by-product of language processing (e.g., as a post-lexical simulation), functionally “redundant” and irrelevant to the efficient semantic comprehension (Mahon and Caramazza, 2008; Lotto et al., 2009). Crucial in this sense is to demonstrate whether embodied processes are automatic and universal mechanisms of linguistic processing, or they are shaped by our sensory, motor, emotional, and linguistic experience.

## L1 and L2 Grounding in Bodily and Linguistic Experience

Fundamental to the hypothesis of language grounding in sensorimotor processes, is the assumption that linguistic and sensorimotor experience develops with similar timings in early developmental stages. While this is mostly the case for a first language (L1), typically it is not for second language (L2) late learners who do not grow up as bilinguals (MacWhinney, 2005). Pulvermüller (1999) showed that during first language acquisition a strong bond between context, sensory-motor experience and language is established. During the so-called “babbling phase” (6–12 months old) perceptual sensitivities of a child “learn” to reflect the tuning of the native language (Werker and Tees, 1999; Pulvermüller and Fadiga, 2010).

Subsequently, word learning is often accompanied by a direct association to objects, actions and properties of their referents in the environment (Carey and Bartlett, 1978; Bloom, 2000; Zwaan and Madden, 2005). These sensory and motor experiences become reactivated when the child and later the adult, encounters the word. In addition, in daily life action verbs often co-occur with bodily movements or visual sensations, contributing to strengthening the link between sensorimotor programs and linguistic concepts (Vukovic and Shtyrov, 2014). Moreover, children also learn the syntactical construction allowing transferring concrete experiences to an abstract meaning, such as in “tell them a story”. This would be associated with a speech motor program and support embodiment effects when processing metaphors and abstract language (Zwaan and Madden, 2005).

Thus, while L1 is acquired in a strong connection to bodily sensations and motor programs and is used daily, L2 is usually learned through symbol manipulation, often based on L1 through analogy or translation and is used in specific settings (school, work, and reading). However, new concepts might borrow sensorimotor groundings from previously grounded symbols (Symbol Grounding Theory, Harnad, 1990). In this view, L2 concepts would acquire their sensorimotor grounding from the equivalent L1 concepts. If so, typical paradigms of embodied cognition tested in L2 speakers would be expected to produce similar effects, although possibly with differences in magnitude and/or the time-course.

## Testing the Language Grounding Hypothesis in L2

Evidence directly addressing the hypothesis of language grounding from an embodied cognition point of view is still limited. The studies discussed in this section are summarized in Table 1.

**Table 1:** Summary of the reviewed studies on grounding effects in L2.

Nr	Study	Sample	Proficiency measures	Methods	Stimuli	Task	Embodied measures	Results
1	Foroni, 2015	L1 = Dutch L2 = English Tested in L1 and L2	Self-reports	Facial EMG	L2 and L1 affirmative and negative sentences in the first person singular describing emotional expressions involving the zygomatic muscle (Relevant predicates – *to smile, to laugh, to grin*) or not (Irrelevant predicates – *to frown, to cry, to whine*) (*I am smiling* vs. *I am frowing*).	To classify images of arrows as to the direction the arrow was pointing to (left or right) after reading the sentences.	Mean facial electromyographic response for the zygomatic major muscle	Motor effects in both in L1 and L2, no simulation in the negative sentences in L2
2	Dudschig et al., 2014	*Ex1.* L1 = German L2 = English *Ex2.* L1 = German L2 = English *Ex3.* L1 = German L2 = English Tested in L1 and L2	N/A	Behavioral	L1 or L2 words referring to entities with a typical location (*star, mole*) or to an emotion (e.g., *happy, sad*)	Vertical Stroop paradigm	Reaction times	L2 automatically triggers the language-action compatibility effect (ACE), both for implicit location and emotion words. Words referring to positive emotions facilitated upward, and words referring to negative emotions facilitated downward responses.
3	Buccino et al., 2017	L1 = Italian L2 = English Tested in L2	CEFR scale (Common European Framework of Reference for Languages: Learning, Teaching, Assessment).	Behavioral	36 English nouns of graspable (*leaf*) or non-graspable (*fog*) objects and 36 pictures of graspable or non-graspable objects; 36 pseudo-words (*leat*) or scrambled images	Sensibility judgment	Reaction times	Slower RTs for processing of graspable items as compared to non-graspable ones, thus similar modulation of motor responses in L1 and L2
4	Parker Jones et al., 2012	L1 = English L2 = English Tested in L1 and L2	A lexical decision test [Psycholinguistic Assessment of Language Processing in Aphasia (PALPA; Kay et al., 1992)]; responses for letter and category fluency (Grogan et al., 2009); a color-word version of the Stroop task (Stroop, 1935)	fMRI	pictures of familiar objects, written object names, pictures of non-objects, and Greek letters/symbols	1. Semantic decision task: matching words and pictures; 2. reading words aloud or naming pictures	Whole-brain analysis	Due to higher demands in L2, higher activation in dorsal precentral gyrus, pars triangularis, pars opercularis, superior temporal gyrus, and planum temporale
5	De Grauwe et al., 2014	L1 = Dutch Tested in L1 L1 = German L2 = Dutch Tested in L2	The online version of the Dutch LexTALE test (Lemhöfer and Broersma, 2012); Score > 67.50%	fMRI; Behavioral	96 Dutch–German morphologically simple verbs (48 cognates and 48 non-cognates; 24 motor-related and 24 not motor-related each) and 48 Dutch morphologically complex verbs (with motor or non-motor simple cognate as stem); 24 pseudo-words (8 complex, 16 simple)	Lexical decision task (word vs. non-word)	Region-of-interest (ROI) and whole-brain analyses	Both L1 and L2 words issue motor-related activations in motor and somatosensory regions.
6	Vukovic and Shtyrov, 2014	L1 = German L2 = English Tested in L1 and L2	Self-reports; The online version of the English LexTALE (Lemhöfer and Broersma, 2012)	High-density EEG	Prime Word Language (L1 vs. L2), Probe Language (L1 vs. L2): 270 action words, 135 in L1 (e.g., *kratzen, spucken, springen*) and 135 in L2 (*drawing, chewing, running*).	Passive reading task	ERD of the mu rhythm	An early event-related desynchronization (ERD) of the mu-rhythm is present in both L1 and L2, being stronger for L1.
7	Vukovic and Williams, 2014	L1 = Dutch L2 = English Tested in L2	Self-ratings; Age of acquisition; Number of years in the UK	Behavioral	24 sentences containing interlingual English-Dutch homophones, suggesting certain distance relations; 24 color image pairs	Sentence-picture matching task	Reaction times	L1 meanings are automatically activated and automatically simulated when reading L2 homophone sentences.
8	Rüschemeyer et al., 2006	L1 = German Tested in L1 L1 = Russian L2 = German Tested in L2	Self-reports; Performance during the experiment	fMRI	96 short German sentences using transitive verbs in the imperfect passive form, in each of the 4 conditions: correct, syntactically incorrect, semantically incorrect, filler	Violation paradigm (sentences with a syntactic, a semantic, or no violation)	Whole-brain analysis	Both L1 and L2 speakers engage language- and motor-related brain regions, the activation being higher for L2 speakers.
9	Gianelli et al., 2018	L1 = German L2 = English Different samples tested in L1 and L2	Self-reports	TMS	Pairs of sequentially presented pictures (tool + tool-oriented hand action) and words (tool noun + tool-action verb). Linguistic stimuli were presented in German (Experiment 1) and English (Experiment 2).	Passive task Stimulation of the primary motor cortex at 5 different timings	Motor-evoked potentials MEPs	Same MEPs modulation in L1 and L2, at 150, 350 and 500 ms after action verb onset


At the behavioral level, a few studies seem to support the idea that during L1 and L2 processing of action-related verbs and nouns, sensorimotor information is similarly activated, though probably only partially (Foroni, 2015). Using a Stroop paradigm, Dudschig et al. (2014) found that L2 automatically triggers action-sentence compatibility effects both for implicit location words (e.g., roof) and emotion words (e.g., happy) in an adapted version of the classic paradigm (ACE; Glenberg and Kaschak, 2002). This paradigm typically employs sentences implying a movement either away from the body or toward the body, such as “You gave the pizza to Andy”, or “Andy gave the pizza to you.”. Participant judge the sensibility of this sentences responding with a movement away/towards the body. In this design, reading times are typically faster in presence of a match between response movements and direction implied by the linguistic stimuli (Lachmair et al., 2011). Since the ACE effect presupposes contextual coupling of language and sensorimotor processing, the results by Dudschig and colleagues provides additional evidence for reactivation of experiential traces of these associations (Glenberg and Kaschak, 2002).

Buccino et al. (2017) recently replicated in L2 the typical motor system modulation induced in L1 by nouns referring to graspable objects as compared to non-graspable ones (Marino et al., 2014). In a go-no go paradigm, motor responses of Italian participants with very good English proficiency showed a significant modulation similar to previous results in L1.

At the neural level, Parker Jones et al. (2012) studied brain activations in native and non-native English speakers with high English proficiency while reading words out loud and during a word-picture matching task. Stimuli included written object names, familiar objects, pictures and symbols. Their study demonstrated that, in comparison to native speakers, non-native English speakers had higher levels of activation in temporal and frontal areas, supposedly because foreign language might need more effort while being retrieved and articulated (McDonald, 2006). Similar results in language as well as in motor-related brain regions for L2 speakers were also reported by Rüschemeyer et al. (2006). Their fMRI experiment based on a violation paradigm conveyed an increased activation of left inferior frontal gyrus as a reaction to a semantic violation in both L1 and L2 speakers, consistently in both auditory and written stimuli. Thus, both L1 and L2 speakers engaged the same cortical network to process language, but with higher activations for L2 speakers.

In the same vein, De Grauwe and colleagues used a lexical decision task showing in fMRI that L2, as well as L1, semantic representations can produce activations to simple motor verbs in motor and somatosensory regions (De Grauwe et al., 2014). Thus, embodiment effects with simple verbs were present with both L1 and L2.

One might argue, though, following Vukovic and Shtyrov (2014), that fRMI does not possess enough temporal resolution to investigate the time-course of linguistic processes and might only show secondary post-comprehension processes (Toni et al., 2008). In the specific case of comparison of L1 and L2 processing, it might thus hinder differences in temporal dynamics of the two processes.

Data collected using M/EEG or focal Transcranial Magnetic Stimulation (TMS) might overcome this limitation but the available evidence is still insufficient. Vukovic and Shtyrov (2014) presented a crucial quantitative difference between L1 and L2 in an equally rapid (from 150 ms on in both languages) motor-cortex involvement. The reported effect, in fact, was stronger for native speakers. Recent TMS results from our laboratory, showed the same modulation of cortical excitability following presentation of pairs of action words in both L1 and L2 (Gianelli et al., 2018, preprint). Notably, the direction and timing of this modulation was comparable to the one induced by visual action processing, pointing to a shared system of action semantics.

## Grounding of Abstract Concepts in L1 and L2

Evidence on L2 grounding effects induced by abstract words is particularly scarce, possibly as a result of the available contradicting evidence of embodiment in L1 for abstract linguistic concepts (Borghi et al., 2017). A large amount of abstract vocabulary has been believed to carry a large emotional load, and several studies have shown that embodiment is also possible through emotion, not only through motion (Moseley and Pulvermüller, 2014; Baumeister et al., 2015; Moseley and Pulvermüller, 2018; see Pulvermüller, 2013 and Moseley et al., 2015 for review). Interestingly, in contrast to the belief that abstract words in both languages are learned relatively later than concrete ones (Chomsky, 1965; Clark and Paivio, 1991), there is evidence that emotional abstract words in L1 are learned much earlier even than concrete concepts, in an immediate interchange between the babies and mother or the caregiver (Kousta et al., 2011). A recent study about abstract vocabulary development found that children mostly learn abstract concepts at the age between 6 and 10, but valenced words are learned quite early at the age of 7 or 8 (Vigliocco et al., 2017). In another study, adults were asked to evaluate the age of positive, negative and neural abstract words acquisition (Ponari et al., 2017). Notably, positive and negative (valenced) words were acquired earlier than neutral ones.

The semantic aspects of abstract words might depend much more on the context in which they were learned (Williams and Cheung, 2011). Thus, speakers of different languages might have different emotional connotations of one word (Eilola and Havelka, 2011). Several studies have revealed higher emotional involvement during L1 processing: for instance, that it is easier to swear in a non-native language (Harris et al., 2003; Dewaele, 2004). Self-rated L2 proficiency significantly predicted perception of emotional force of taboo words. Negative abstract words elicit stronger arousal in L1 compared to L2, especially when L2 was acquired in an adult age. On the other hand, evidence from an eye-tracking study by Sheikh and Titone (2016) suggests an emotional grounding only for positive words: only positive, and not negative, words were in fact read more quickly than neutral words in L2.

## Theories Of L1 and L2 Processing

From the reviewed evidence, an explicit sensorimotor and emotional grounding approach to L2 has so far been taken only by a limited number of studies. However, it is worth noting that evidence on L1 and L2 outside this particular approach is much more extensive and has produced several competing models and hypotheses regarding the neural bases of L1 and L2. It is worth briefly reviewing them in order to discuss whether and how the embodied cognition evidence might support the existing models.

According to the so-called differential hypothesis (Ullman, 2001), different neural mechanisms support L1 and L2 processing. This hypothesis is supported by empirical evidence from experiments of selective recovery with bilingual patients (Aglioti and Fabbro, 1993; Mehrpour et al., 2014). In bilingual aphasic patients recovery of linguistic abilities first takes place in only one of the languages and later in the other one. In contrast, the neural convergence hypothesis (Green, 2003) claims that acquisition and processing of L2 is based on the same neural mechanisms as L1 (Perani and Abutalebi, 2005; Abutalebi, 2008) and only differs as to the age of acquisition, the task and the level of proficiency (Liu and Cao, 2016). The more fluency L2 speakers gain, the more similarity there is between L2 and L1. Most evidence agrees on the common neural bases of L1 and L2 processing (Perani and Abutalebi, 2005), with the latter possibly having a more complex structure of activation. For example, several studies found divergence in time-course and in activation topography between semantic and phonological tasks between L1 and L2 (Marian et al., 2003; Pillai et al., 2003). A connected issue is the question whether lexical candidates from both languages are activated and whether they are stored in one integrated lexicon or different ones (Dijkstra and Van Heuven, 2002), constituting the so-called language non-selective vs. language selective access hypotheses. The Bilingual Interactive Activation (BIA) model (Dijkstra et al., 1998) is a language comprehension model that claims that lexical access is non-selective and lexical items are stored in one unique mental lexicon. This model posits a non-selective bottom-up processing at the level of the following language nodes: features, letters, words, language. On visual input onset, features inhibit those letters for which these particular features are absent and excite those letters for which the features are present. The letters, in their turn, inhibit or excite words if they are present or absent, respectively, and words inhibit each other irrespective of the language. Finally, the corresponding language nodes are also activated. An important part of this model is a language selective top-down inhibition, meaning that language nodes send inhibitory signals to all the words in another language. L2 words also activate the L1 words that activate the L1 language node, which, in turn, inhibits L2 words. Thus, lexical candidates from both languages are activated. The more recent BIA+ model extends these assumptions from orthographic representations to phonological and semantic representations (Dijkstra and Van Heuven, 2002). Multiple empirical findings support this hypothesis: neighborhood density effects within and between languages (Van Heuven et al., 1998; Dijkstra and Van Heuven, 2002), shifting neighborhood effects across an experiment, masked orthographic priming effects in within and between languages in bilinguals (BijeljacBabic et al., 1997), language switching effects in lexical decision task (Von Studnitz and Green, 1997). Today, most scientists agree that bilingual speakers activate both languages (Costa et al., 2017). Recent evidence suggests an automatic activation of the native language while processing L2: Vukovic and Williams (2014) tested Dutch participants hearing English sentences containing interlingual homophones suggesting certain distance relations and instructed them to respond to pictures matching or mismatching this distance. Their results showed that participants automatically activate L1 meanings when reading L2 homophone sentences.

According to these models, if language is indeed embodied and grounded in sensorimotor (and possibly emotional) processes, then a similar engagement of these systems should be expected regardless of the use of L1 or L2. However, the time course might differ, with later L2 effects (Hahne, 2001; Spalek et al., 2014). These effects would be compatible with the hypothesis of L2 effects being mediated by an L1 motor resonance (Foroni, 2015).

Similar temporal dynamics, on the other hand, would support effects directly produced by L2 linguistic stimuli, not mediated by L1. This would for instance be in line with the Revised Hierarchical Model (RHM) (Kroll and Stewart, 1994; Kroll et al., 2010), a language production model in favor of the selective activation hypothesis. This model postulates that, depending on the context, bilinguals activate either the L1 or the L2 lexicon selectively. This model postulates that bilingual memory is a hierarchical system with two levels of representation: lexical store and the conceptual level. Between the lexical representations in L1 and L2 as well as between the lexical representations in each language, there is supposed to be a level of conceptual representations, the latter being shared between the languages in most cases, except for more abstract words. This model predicts the development of these links along with L2 acquisition. In the beginning phase, the connections between the lexical level in the L2 and the conceptual level are very weak and more affected by form-related variables, and strengthen as the individual gains a higher L2 proficiency. Numerous cross-language semantic priming studies provide empirical evidence confirming the RHM (Kroll and Borning, 1987; De Groot and Nas, 1991). A critical important assumption of the RHM is that both the age of L2 acquisition and the L2 learning context – a formal or a natural setting - modulate the effects of semantics on L2 (Ferré et al., 2006). Later studies suggest that there is not only a link between the L1 and L2 semantics, but specifically to those aspects of L2 semantics, which were active during the learning phase (Williams and Cheung, 2011). In this sense evidence collected from embodied cognition paradigms, which take into account the timings and proficiency of both sensorimotor and linguistic learning, might provide crucial support for this assumption.

## Conclusion

It is evident that approaching L1 and L2 processing from an embodied point of view is a new and complex area of research. On the one hand, L2 poses a challenge to strong views of embodied cognition: if linguistic concepts are deeply rooted in sensorimotor processes, then this should be true regardless of the use of L1 or L2. On the other hand, the same assumption of a strong link between sensorimotor experience and linguistic concepts, would predict differences between L1 and L2 grounding based on their age of acquisition.

Furthermore, when approaching L1 and L2 existing competing models should be taken into account and integrated models should be produced including evidence based on the embodied and grounding hypothesis. In this sense, these models should take into account several parameters, including localization, magnitude and timing of the embodied effects.

The use of methodologies with high temporal resolution, such as M/EEG, or allowing focal brain stimulation (TMS), will be crucial for disentangling the time course of effects in L1 and L2. In addition, behavioral measures collected in various tasks, will allow a more complete view of how L1 and L2 linguistic concepts are shared or differentially activated. The possible task-dependency of these effects– an issue already recognized in the embodied language literature – has to be even more carefully addressed when testing L2 as it might either support or conflict with existing strategies used for language acquisition and/or memory retrieval. Such an improved methodological approach would help in clarifying the different functional role of language grounding in L1 and L2.

In the same vein, researchers should focus on formalizing the way linguistic experience (e.g., age of acquisition, proficiency, L2 acquisition strategies) can be factored into the experimental design. Finally, the advancement of research on embodiment in L2 will necessary profit from the extension of the stimuli from single nouns or verbs to more complex sentences, as well as towards the use of abstract and emotional stimuli.

## Author Contributions

CG and KK wrote and approved the final version of this manuscript.

## Conflict of Interest Statement

The authors declare that the research was conducted in the absence of any commercial or financial relationships that could be construed as a potential conflict of interest.
